# Use of Self-Organizing Map to Analyze Images of Fungi Colonies Grown from *Triticum aestivum* Seeds Disinfected by Ozone Treatment

**DOI:** 10.1155/2012/865175

**Published:** 2012-10-16

**Authors:** Thierry Marique, Olivier Allard, Martin Spanoghe

**Affiliations:** ^1^Centre pour l'Agronomie et l'Agro-Industrie de la Province de Hainaut (CARAH), 11 rue P. Pastur, 7800 Ath, Wallonie, Belgium; ^2^Haute École Provinciale de Hainaut-CONDORCET, 17 Chemin du Champ de Mars, 7000 Mons, Wallonie, Belgium

## Abstract

We submitted to ozone treatment *Triticum aestivum* (common wheat) seeds severely contaminated by fungi. Fungi colonies developed when seeds were placed over malt agar medium in Petri dishes; *Fusarium sp*. and *Alternaria sp*. were identified. However, conventional colonies counting did not allow a clear assessment of the effect of ozone disinfection. We thus used self-organizing maps (SOMs) to perform an image analysis of colonies surface area that clearly showed a significant disinfection effect on *Fusarium sp*.

## 1. Introduction

Cereal seeds must meet several quality criteria, in particular good germinative potency (85% after 8 days) and sanitary state (absence of* Claviceps purpurea*). More generally, low contamination from fungi such as *Fusarium, Septoria,* or *Alternaria* is advisable to obtain good performance after seeding. Moreover, fungal contamination of seeds can lead to mycotoxins production such as aflatoxin B1, B2, G1, G2, M1, ochratoxin A, zearalenone, and fumonisin [1]. It is thus common practice to treat seeds with antifungal molecules, but this leads to evident environmental problems and even to concern about safe disposal of seed packaging. Alternative solutions will likely become widely spread in the future and must be investigated.

Among them, ozone is wellknown for its efficiency as a surface disinfection agent, especially as it leaves no toxic residue once converted into oxygen. It has also been proved active against mycotoxins [2]. Stability of pure gaseous ozone is about 3 days at 20°C and falls to about half an hour in practical conditions [3]. As a potent oxidative agent, it is efficient against both gram− or gram+ bacteria, fungi, viruses, protozoa, and also bacterial or fungal spores. Apart from its use in water sanitation, it has also been proposed to lower pathogen microbial contamination on diverse plant seeds or bulbs (*Triticum sp.*, *Brassica sp.*, *Zea mays, Hordeum sp., Phaseolus sp., Helianthus sp.*,…) [1, 4, 5]. A decisive advantage of ozone is its low toxicity on seed germs which allows to preserve excellent germination performance.

Several methods can be used to assess ozone disinfection efficiency. Apart from direct surface visual inspection, one can also wash a seed lot and microscopically inspect the solution collected therefrom to identify foreign bodies (sclerotes, spores,…). One can also incubate seeds over an *ad hoc* culture medium for a period of 5 to 7 days in suitable temperature and lighting conditions and observe colony development [6, 7]. Fungi identification is then performed from both macroscopical and microscopical characteristics.

We used such an approach to assess ozone surface disinfection performance on *Triticum aestivum* (common wheat) seeds. Samples heavily contaminated by *Fusarium *were incubated over malt agar medium in Petri dishes. Fungi colonies were counted and identified. However it appeared to us that a more thorough scrutiny of the results was necessary. While colonies numbering did not point to a strong effect of ozonation, colony surface area was clearly reduced.

We thus used self-organizing maps (SOMs) to analyze surface colony area. SOMs are a particular kind of artificial neural networks that learn to classify input vectors (here pixel RGB intensity data) according to how they are grouped in the input space. They differ from competitive layers in that neighboring neurons in the self-organizing map learn to recognize neighboring sections of the input space. Thus, SOMs learn both the distribution (as do competitive layers) and topology of the input vectors they are trained on [8]. SOMs have been used successfully to perform segmentation of complex images, in particular from diverse food products such as cookies, fried chips, or potatoes [9–12]. In the present case, several SOMs were trained to mimic color topology of the different fungi colonies. This allowed to discriminate quickly and easily between different fungi species while dodging the necessity to remove image background beforehand.

This approach showed a significant inhibition of *Fusarium* growth after ozone treatment, while seeds retained a germinating capacity over 95%. Ozonation thus is qualified as an efficient treatment for seed surface disinfection, while SOM use allows to fine tune image analysis of fungi colonies.

## 2. Material and Methods

*Triticum aestivum* seeds were obtained from Jorion SA, Hacquegnies, Belgium. A particular sample from cv *Louisart* (moisture 14, 3%) was selected for its known huge contamination by *Fusarium*. 

Ozone was produced by corona discharge using a benchtop generator (C-Lasky series Ozone Generator C-L010-DT, AirTree Technology Europe Co., Germany), with a maximal production of 2 g/h when fed with atmospheric air. Both air flow and corona discharge power could be adjusted. 

Seed samples (60 g each) were placed in a cylindrical vessel (15 cm in diameter). They were exposed for a known time to ozone treatment by percolating gas flow (5 L/min, 0,8 mg ozone/L) from the generator through a perforated bottom. The vessel was shaken every minute during operation to ensure homogeneous contact between gas and grain surface. 

**Figure 1:**
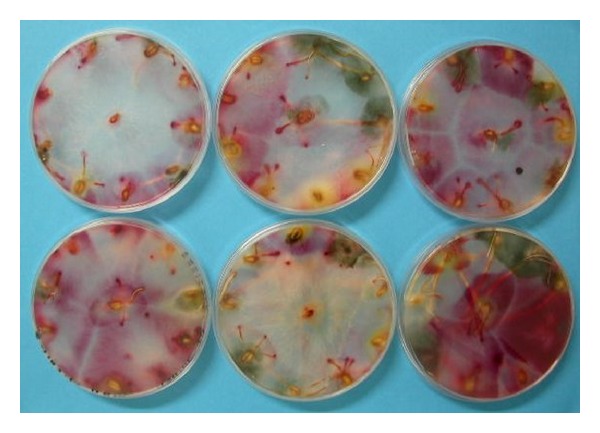
Image of a set of six Petri dishes bearing colonies of both *Alternaria sp. *(green) *and Fusarium sp. *(rosy). Yellowish germinated grains can be observed.

Petri dishes filled with Malt agar (Biokar diagnostics, France) were used for fungi detection. For each assay, a total of 300 seeds were settled in 30 Petri dishes. In a particular Petri dish a seed was settled in the center, and nine others were settled equally spaced on the periphery (Figure 1). Control samples were used, that is, untreated seeds and seeds commercially treated with *Celest *(fludioxonil, Syngenta Crop Protection NV, Belgium). Dishes were incubated for 7 days at 20°C, in the dark. Colonies were counted and inspected both macroscopically and microscopically to identify fungi species, as detailed in previously published methods [6, 7]. Colonies count was performed seed by seed. Some seeds displayed no fungal growth. Most displayed growth of a single fungal species and were counted one for that species. Some displayed growth of several species and were counted one for every species found.

Petri dishes were then positioned over a sky blue background in a lighting cabinet (GTI MiniMatcher, GTI graphic technology, Newburgh, USA) equipped with two 6500°K daylight-balanced fluorescent lamps. Cabinet sides were lined with mirrors to minimize border shadows.

A digital camera (Nikon Coolpix 4500) was used to take images (Figure 1) in jpeg format (640 × 480 pixels). White balance correction was activated. Images (8 bit) were then imported into MATLAB 2011 software (The MathWorks, Inc., Natick, USA).

**Figure 2:**
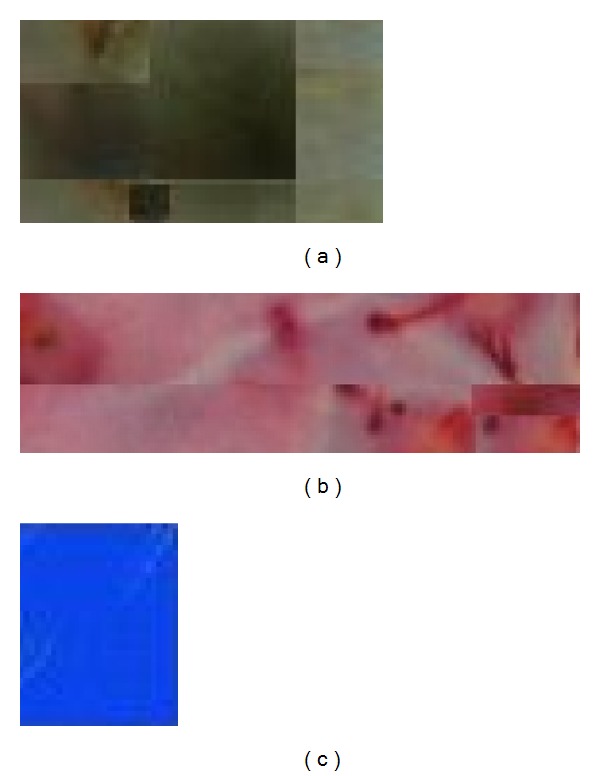
Composite images used to train, respectively, *Alternaria* SOM (a), *Fusarium* SOM (b), or background SOM (c).

Three bidimensional SOMs (hexagonal networks) of 5 × 10 neurons each were created using the MATLAB *Neuron Network Toolbox*. The networks were limited to two dimensions only to match the flat shapes of the clusters of RGB values from the two fungi colonies types (*Alternaria sp.* and *Fusarium sp.*) and of the image background. These SOMs were trained using three composite images created by sampling a representative set of regions from colonies of *Alternaria sp.,* from *Fusarium sp.* Colonies, or from the image background (Figure 2). 

**Figure 3:**
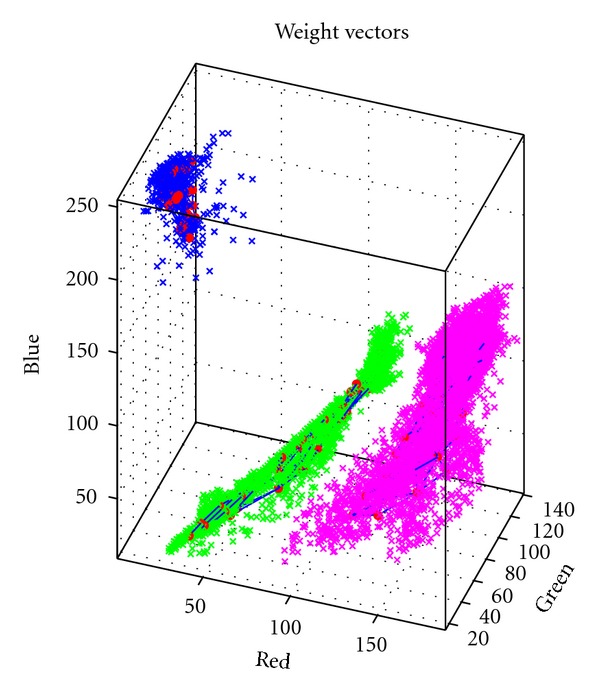
Three SOMs (represented as red dots connected by blue lines) fitted to three training sets of RGB color coordinates (green for* Alternaria sp.*, magenta for *Fusarium sp.*, and blue for image background).

This allowed to account for the whole complex set of color shades present in the colonies. Over training iterations, the SOM progressively unfolded to match the shapes and topologies of the color pixel distributions (Figure 3).

**Figure 4:**
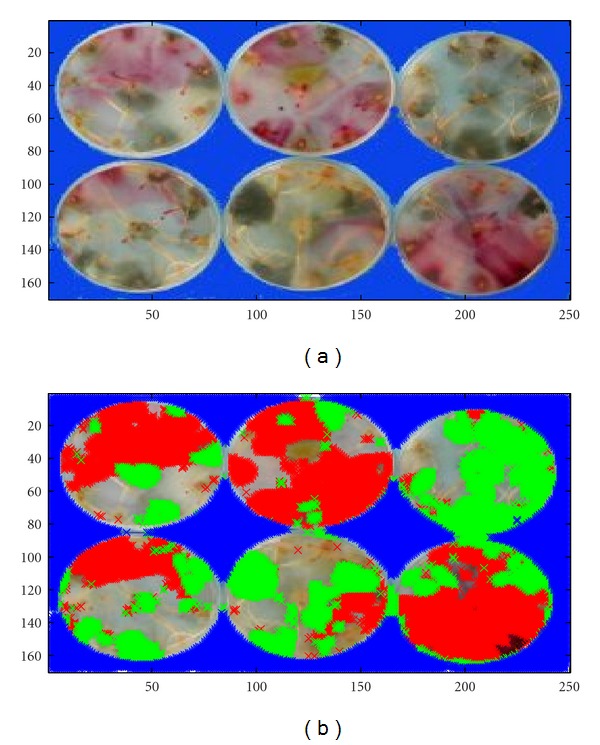
Photograph of Petri dishes (a) and the same with superposed masks for colony types (b): green mask for *Alternaria sp.*, red mask for *Fusarium sp.*, and blue mask for image background.

The trained SOMs were then used to perform segmentation of images (Figure 4). For each pixel presented to a SOM, a single winning neuron would respond as being closest to these particular pixel RGB coordinates. Euclidean distance between the winning neuron and pixel coordinates was then computed and used to decide whether the considered pixel was part of an *Alternaria* colony, of a *Fusarium* colony, or of none of them (thus being background). In either colonies, ratio of the number of pixels to the total pixel number of a Petri dish would finally provide an estimation of colony surface percentage.

Seed-germinative capacity was evaluated by testing 400 seeds for each treatment, placed into four essay boxes (100 seeds each).

Essay boxes are 21 cm long, 12 cm broad, and 8 cm high, with a cover to guard against water evaporation. Germination substratum is prepared by mixing 87% dry white sand with 13% water. This substratum is then disposed in the bottom of the boxes, with a constant height of 2 cm (about 820 g per box).

In each box, 100 equally spaced small holes are perforated through the sand by way of a nailed plank (nails protrudes 1 cm). Each hole then receives a seed, and covered boxes are first incubated for 7 days at 6-7°C in a refrigerated cabinet. They then stay for 8 days at 22°C in a temperature-regulated cabinet lighted with 12 neon tubes (PHILIPS TL-D58W/54-765), with a cycle of 12 day hours and 12 night hours. Boxes are finally collected, and germinated seeds are counted.

## 3. Results

*Triticum aestivum* seeds, variety of *Louisart*, were exposed to ozone treatment for different durations and then incubated over Petri dishes for 7 days. Fungi colonies developed that were, respectively, identified as *Fusarium sp.* and *Alternaria sp*. (Figure 1).

**Figure 5:**
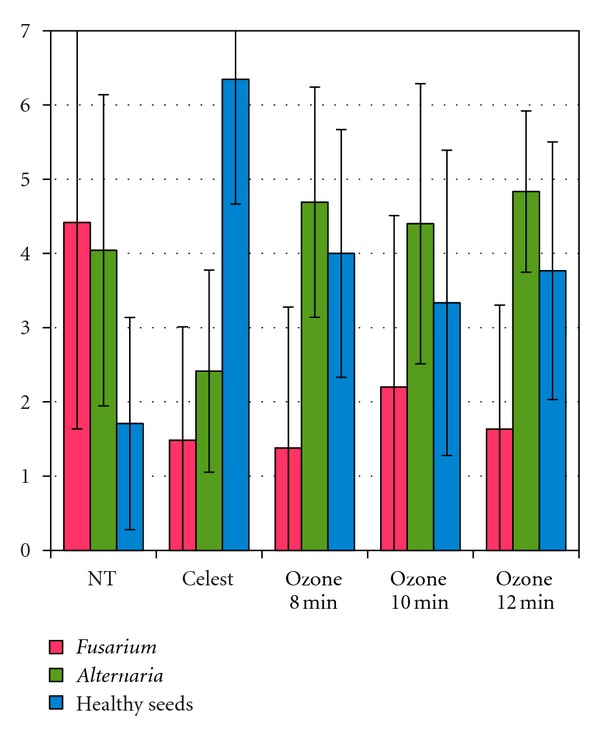
Number of *Fusarium sp. *colonies, of *Alternaria sp.* Colonies, or of healthy seeds per Petri dish, for seeds nontreated (NT), treated with *Celest*, or treated with ozone for 8, 10, or 12 minutes.

Colonies number was counted. As shown in Figure 5, results yielded very poor information due to large dispersion. The only significant effect was a higher number of healthy seeds when treated with *Celest*. However, no significant effect could be seen for either *Alternaria* or *Fusarium* counts.

A more thorough inspection of Petri dishes however hinted at the existence of an effect of ozone treatment on colony surface. To test this, we measured colony surface by way of image analysis through a SOM methodology.

**Figure 6:**
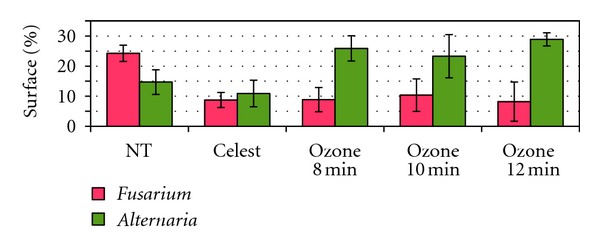
Surface percentage of *Fusarium sp. *colonies or of *Alternaria sp.* colonies per Petri dish, for seeds nontreated (NT), treated with Celest, or treated with ozone for 8, 10, or 12 minutes.

As shown in Figure 6, seed treatment with *Celest* led to significantly smaller *Fusarium* colonies (about threefold); no significant effect was observed on *Alternaria* colonies. Seed treatment with ozone also led to significantly smaller *Fusarium* colonies (about threefold). However, a significant effect was observed on *Alternaria* colonies, with surfaces about twofold greater after treatment.

**Figure 7:**
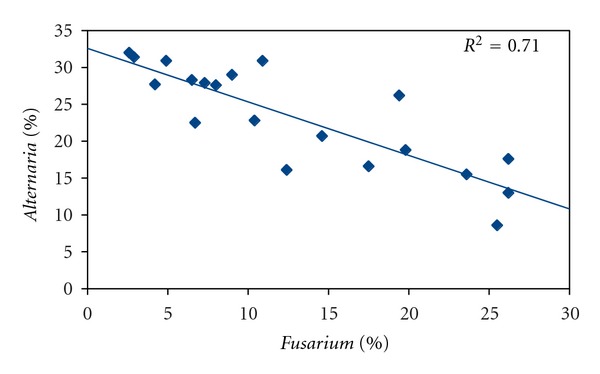
Petri dish surface percentage occupied by *Alternaria sp.* colonies as a function of Petri dish surface percentage occupied by *Fusarium sp. *colonies, for seeds treated with ozone.

Figure 7 shows the linear negative correlation between the surfaces of *Fusarium* and *Alternaria* colonies, for seeds treated with ozone. We see that reduced *Fusarium* surface results in increased *Alternaria* surface, with a correlation coefficient of 0.71.

We also performed germination assays with seeds from different treatments. All seeds retained the same germinative capacity than control, that is, over 95%, with the exception of seeds treated with ozone for more than 10 minutes in our experimental conditions, where germinative capacity was lowered to 90%. 

## 4. Discussion

The use of SOM to operate segmentation of fungi colonies on Petri dishes images proved to be very efficient, as was already the case in earlier studies [9–12]. We were able to obtain fast and easy image treatment, with very good segmentation between *Fusarium sp.* and *Alternaria sp.* colonies. Whole colony surfaces were selected, due to the SOM taking into account the whole range of color shades for the two types of fungi colonies.

As previously described [1, 13], we observed an effect of gaseous ozone treatment to reduce fungi seed contamination without germinative impairment, except for prolonged ozone treatment. However, our SOM method to measure surface occupation by colonies allowed to obtain more precise and detailed data, which led to better understanding of treatment effects. First, a simple count of colonies number showed no significant decrease except for *Celest* control. By colony surface analysis, we also observed a decrease of both *Fusarium* and *Alternaria* growth after *Celest* treatment. This product is thus efficient on both fungi species. Ozone treatment had a different effect, effective on *Fusarium* only: it led to a decrease in *Fusarium* surface occupation (the number of *Fusarium* colonies is not decreased, but colonies are smaller) and to an increase in *Alternaria* surface occupation. Thus, reduced growth of *Fusarium* allows *Alternaria* to occupy more surface on Petri dishes. Two reasons can be advanced to explain the low toxicity of ozone on *Alternaria*. *Alternaria* could be less susceptible to ozone toxicity, due to a more efficient spore resistance. Also, *Alternaria* could be settled deeper into seed tegument and thus less exposed to ozone attack.

## 5. Conclusions

From this, we conclude that ozone is a more selective disinfectant than *Celest*, acting efficiently on *Fusarium*. Enhanced growth of *Alternaria* is attributed to lower competition after *Fusarium* was killed by ozone treatment. It would be of interest to use this method to obtain detailed data about seed disinfection for a greater variety of fungi contaminants.

Image treatment of colonies by SOM segmentation proved an efficient method. There is still to test its limits in case of colonies presenting closely matching color shades.
